# Naïve adaptive immune receptor repertoires in celiac disease assessed by machine learning; impact of the HLA-DQ2.5 allotype on the TCR repertoire

**DOI:** 10.1007/s00251-026-01412-3

**Published:** 2026-08-27

**Authors:** Aengus Officer, Ida Lindeman, Milena Pavlovic, Christin Lund-Andersen, Eivind Ness-Jensen, Geir Kjetil Sandve, Ludvig M. Sollid

**Affiliations:** 1 Norwegian Coeliac Disease Research Centre, University of Oslo, Oslo, Norway; 2 Institute of Clinical Medicine, University of Oslo, Oslo, Norway; 3 Department of Immunology, Oslo University Hospital – Rikshospitalet, Oslo, Norway; 4 Department of Informatics, University of Oslo, Oslo, Norway; 5 Department of Tumor Biology, Institute for Cancer Research, Oslo University Hospital – The Norwegian Radium Hospital, Oslo, Norway; 6 HUNT Research Centre, Department of Public Health and Nursing, NTNU, Norwegian University of Science and Technology, Levanger, Norway; 7 Department of Medicine, Levanger Hospital, Nord-Trøndelag Hospital Trust, Levanger, Norway; 8 Upper Gastrointestinal Surgery, Department of Molecular Medicine and Surgery, Karolinska Institutet and Karolinska University Hospital, Stockholm, Sweden

**Keywords:** Celiac disease, HLA, T-cell receptors, Machine learning, Naive repertoires, AIRR, B-cell receptors

## Abstract

The adaptive immune receptor repertoire (AIRR) - the collection of an individual’s B-cell and T-cell receptors (BCRs and TCRs, respectively) - encodes cumulative immune history and is shaped by both germline genetics and environmental exposures. Skewed repertoires have been linked to infections, vaccination responses and autoimmune diseases such as celiac disease (CeD) where biased usage of immunoglobulin and T-cell receptor genes reactive to disease relevant antigens has been reported. Motivated by evidence that germline variation, notably human leukocyte antigen (HLA), influences naïve AIRRs and by prior machine-learning studies that classified CeD using naïve BCR AIRR-sequencing (AIRR-seq) data, we applied machine learning analysis to naïve CD4^+^ TCR and naïve BCR AIRR-seq repertoires to test whether repertoire features can distinguish subjects with CeD from controls and to identify drivers of such classification. Naïve CD4^+^ TCR repertoires yielded moderate diagnosis classification, but this signal was largely explained by enrichment of the HLA-DQ2.5 allotype. TCR variable gene frequencies predicted HLA-DQ2.5 status with high accuracy and controlling for HLA-DQ2.5 abolished TCR-based diagnosis classification. In contrast, naïve BCR repertoires could not be used to classify CeD. Overall, our findings show that germline HLA variation significantly affects naïve TCR composition and thereby indirectly facilitates moderately successful CeD status classification.

**Supplementary Information:**

The online version contains supplementary material available at 10.1007/s00251-026-01412-3.

## Introduction

The collection of an individual’s B-cell receptors (BCRs) and T-cell receptors (TCRs) makes up one’s adaptive immune receptor repertoire (AIRR). AIRRs are unique, and their composition is influenced both by genetics and environmental factors. After encountering antigen, B cells and T cells become activated, clonally expand and daughter cells inherit the same receptor as the mother cell. Subsequently, after infection memory cells that are specific to the antigen will confer long-term protection. Therefore, the AIRR offers valuable insight into an individual’s past and current immune state. Skewed AIRRs have been shown in infection settings (Chen et al. 2017; Crowe 2019; Di Niro et al. 2010; Nielsen et al. 2020; Niu et al. 2020) as well as after vaccination (Jackson et al. 2014; Jiang et al. 2013; Strauli and Hernandez 2016), where certain genes for immunoglobulin (IG; BCR) or TCR genes (TR) dominate the repertoire.

This phenomenon of skewed AIRRs has also been observed in autoimmunity (Bashford-Rogers et al. 2018, 2019; Carreno et al. 2015; Hershberg and Luning Prak 2015). A notable example is celiac disease (CeD) where there is biased usage of both IG and TR genes encoding antigen receptors against deamidated gluten epitopes (Dahal-Koirala et al. 2021; Jabri and Sollid 2017; Lindeman et al. 2021; Snir et al. 2017; Steinsbø et al. 2014) as well as a preference for certain IG genes against the autoantigen transglutaminase 2 (Lindeman et al. 2024; Roy et al. 2017). In the case of CeD-specific BCRs, these have been shown to undergo minimal somatic hypermutation (Di Niro et al. 2012; Lindeman et al. 2021; Roy et al. 2017; Snir et al. 2017; Steinsbø et al. 2014), leading to the hypothesis that there are germline signatures in the naïve AIRRs that can make one predisposed to develop CeD. We have recently demonstrated the significant effect of *cis*-acting germline polymorphisms on the AIRRs of naïve T cells and B cells, both in CeD and controls (Lindeman et al. 2026; Officer et al. 2026). In addition, the naïve TCR repertoires were significantly associated with *trans*-effects from human leukocyte antigen (HLA) germline variants (Lindeman et al. 2026), whereby HLA impacts the naïve TCR repertoire through thymic selection. In active CeD, HLA has been shown to influence the TCR repertoire of effector T cells in the periphery through presentation of gluten peptides (Broughton et al. 2012; Dahal-Koirala et al. 2016; Petersen et al. 2014, 2015, 2016; Qiao et al. 2011, 2014) presented by the CeD associated HLA allotypes DQ2.5, DQ8 or DQ2.2 (Sollid 2017).

Given the distinct gene usage frequencies in the naïve TCR repertoires observed in CeD and the impact of HLA on the repertoire, we hypothesized that machine learning approaches could be utilized to classify subjects with CeD versus controls using naïve TCR AIRR-sequencing (AIRR-seq) repertoires, and further to identify factors that drive this classification. Machine learning approaches have been used to predict immune states by AIRR classification in a variety of settings, including in autoimmunity (Shemesh et al. 2021; Yao et al. 2021) and in cancer (Ostmeyer et al. 2019; Xiong et al. 2021). Integration of AIRR-seq data into machine learning models can potentially lead to single diagnostic tests and identification of novel therapy targets (Arnaout et al. 2021). Through leveraging certain common features of adaptive immune receptors, such as shared use of complementarity determining region 3 (CDR3), variable (V), joining (J) or diversity (D) genes, nucleotide or amino acid sequences, machine learning can be used to accurately predict the immune state of an individual.

Multiple machine learning studies have demonstrated highly accurate classification of subjects with CeD using TCR sequences from effector T cells (Foers et al. 2021; Fowler et al. 2023; Yao et al. 2021), but to date no machine learning models have considered the naïve T-cell repertoires in CeD. Machine learning has however been used previously to analyze naïve BCR sequencing data from individuals with CeD and controls, and it was shown to accurately classify subjects with the disease (Shemesh et al. 2021). Using an independent and larger cohort, we aimed to classify the naïve BCR repertoires of CeD subjects and to replicate these previous findings.

Our results reveal that naïve CD4^+^ TCR repertoires of CeD subjects can be distinguished from controls with a moderately high accuracy, with a median area under the receiver operating characteristic curve (AUROC) score of 0.72 using logistic regression models on a V gene frequency encoding of repertoires. This classification was, however, mainly driven by differences in the repertoires related to HLA-DQ allotypes with the CeD group being highly enriched for HLA-DQ2.5^+^ individuals. Indeed, we were able to predict whether an individual carried the HLA-DQ2.5 allotype based solely on the frequencies of variable and joining genes of TRA and TRB in naïve T cells with a high median AUROC score of 0.93. After controlling for the biased frequency of the HLA-DQ2.5 allotype among subjects with CeD, we were no longer able to classify subjects with CeD from controls using the naïve TCR repertoires. Similarly, our models could not classify individuals as CeD or controls based on the naïve BCR repertoires.

## Materials and methods

### Generation of datasets

Our study consists of three published datasets. We used a naïve TCR AIRR-seq dataset (Lindeman et al. 2026) and a naïve BCR AIRR-seq dataset (BCR dataset 1) (Officer et al. 2026), these are deposited at the federated European Genome-Phenome Archive (EGA) under accession numbers EGAS50000001882 and EGAS50000001881, respectively. In addition, we used a second naïve BCR dataset (BCR dataset 2) (Gidoni et al. 2019), which is deposited at the European Nucleotide Archive (ENA) under accession number PRJEB26509 and was previously used in a machine learning model by Shemesh et al. 2021. After processing steps, BCR dataset 2 included naïve BCR AIRR-seq information from 48 subjects with CeD and 44 controls. The TCR dataset and BCR dataset 1 have been utilized in Lindeman et al. 2026 and Officer et al. 2026; respectively. BCR dataset 1 consists of 102 subjects with CeD and 102 controls, while the TCR dataset consists of 103 subjects with CeD and 103 controls. All datasets derive from Norway, and the TCR dataset and BCR dataset 1 originate from the fourth population-based Trøndelag Health Study (HUNT4) in Norway (Andersen et al. 2025; Åsvold et al. 2023; Lukina et al. 2024). This is an area of low migration and a relatively ethnically homogenous cohort. Naïve B and T cells were isolated separately from PBMCs using FACS. Full details for the generation of these datasets can be found in the respective publications.

### Processing of datasets

Full details of the processing of BCR dataset 1 and the TCR dataset can be found elsewhere (Lindeman et al. 2026; Officer et al. 2026). BCR dataset 1 sequences covered the entire variable gene region of the BCR with occasional nucleotide gaps introduced between paired-end reads, while the TCR dataset consisted of sequences covering part of the V gene (from approximately CDR2) to the end of the J gene.

To allow a fair comparison of BCR dataset 1 with BCR dataset 2, we chose to use two different versions of BCR dataset 2 – BCR dataset 2a and 2b. BCR dataset 2a had already been through the pre-processing pipeline using pRESTO and TIgGER as detailed elsewhere (Gidoni et al. 2019). This dataset was further processed using the same steps as done for BCR dataset 1. For BCR dataset 2b we used the raw fastq reads deposited at the European Nucleotide Archive (ENA), under accession number PRJEB26509. We processed the reads using a pipeline as similar to that used for BCR dataset 1 as possible. This was done in an effort to minimize any potential biases that could have been introduced during the data pre-processing steps of the previously generated dataset. All repertoires were additionally filtered to productively rearranged sequences with a minimal junctional length of five amino acids.

### CeD-relevant reference sequences

A TCR sequence reference database was used with TRA (*n* = 2562) and TRB (*n* = 2428) sequences generated from single-cell AIRR-seq of gluten-reactive HLA-DQ2.5-restricted effector memory T cells isolated from peripheral blood and/or intestinal lamina propria from 50 CeD subjects (Dahal-Koirala et al. 2021). Similarly, a BCR sequence reference database consisting of unique heavy and light chain IG sequences generated from single gut plasma cells reactive with a deamidated gluten peptide epitope (*n* = 183) or transglutaminase 2 (*n* = 4311) was also utilized (Lindeman et al. 2021, 2024; Roy et al. 2017; Vaz et al. 2025). These two reference sequence databases formed the basis for our reference sequence-based machine learning encodings.

### Subsets for HLA classification

HLA allotypes for subjects in the TCR dataset had previously been established by exon sequencing of HLA-DQA1 and HLA-DQB1 genes, HLA inference and/or HLA-typing using a One Lambda LABType SSO Class II DQA1/DQB1 typing test as reported (Lindeman et al. 2026). During the machine learning analysis subjects were divided into distinct groups based on their HLA-DQ allotypes. For the purpose of HLA classification into HLA-DQ2.5 versus other-DQ allotypes and for classification based on diagnosis in only HLA-DQ2.5^+^ individuals, if a subject had at least one copy of HLA-DQ2.5, the subject was placed in the HLA-DQ2.5^+^ group and all other subjects were placed within the other-DQ group.

### Modeling approaches

ImmuneML is an open-source ecosystem designed for AIRR machine learning (Pavlović et al. 2021). The platform is fully compatible with AIRR-seq data, and it selects the most accurate machine learning model based on strict assessment. Machine learning was used to classify repertoires either by diagnosis using BCR or TCR datasets, or by HLA-DQ allotype using the TCR dataset as input. TCR repertoires were encoded using TCR gene-usage, CDR3 k-mers, reference-sequences or shared clonotypes in our machine learning analysis. Similarly, BCR repertoires were encoded by BCR gene usage or CDR3 k-mers. Performance of the different models and encodings was assessed using cross validation. Full detailed information on the repertoire analyses conducted can be found in the Supplementary material and methods section.

## Results

**Fig. 1:**
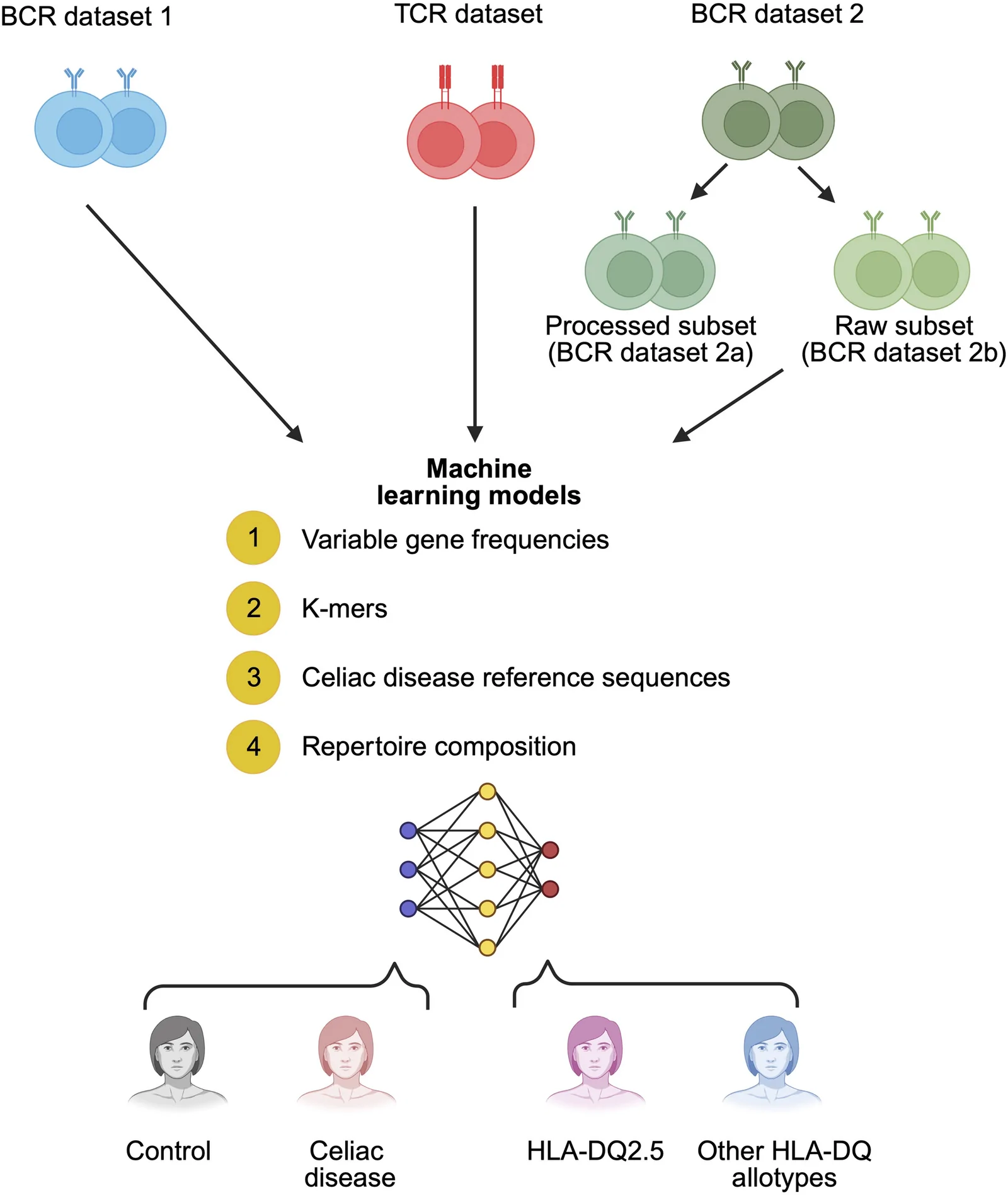
Approach. Three independent naïve AIRR-seq datasets were used as input into different machine learning models. Models include encodings for variable gene frequencies, k-mers, celiac disease-associated reference sequences and repertoire composition. AIRR-seq datasets were classified into either controls and CeD or HLA-DQ2.5 and other HLA-DQ allotypes

Machine learning analysis was conducted using ImmuneML with three separate naïve AIRR-seq datasets as input (Fig. 1); a TCR dataset (Lindeman et al. 2026) and a BCR dataset (BCR dataset 1) (Officer et al. 2026) generated from the HUNT4 study population, and a BCR dataset (BCR dataset 2) previously published by Gidoni et al. 2019 and analyzed by Shemesh et al. 2021. Each dataset contains balanced numbers of CeD subjects and controls. Datasets were separated into training, validation and independent test subsets. Cross-validation was performed to test how well the models generalized. To assess the performance of our models, we chiefly used statistics on the AUROC and the balanced accuracy. Our ImmuneML models tested various features of the AIRRs that were shared among individuals with the same diagnosis or HLA allotype.

### TRV genes assist accurate diagnosis classification

**Fig. 2:**
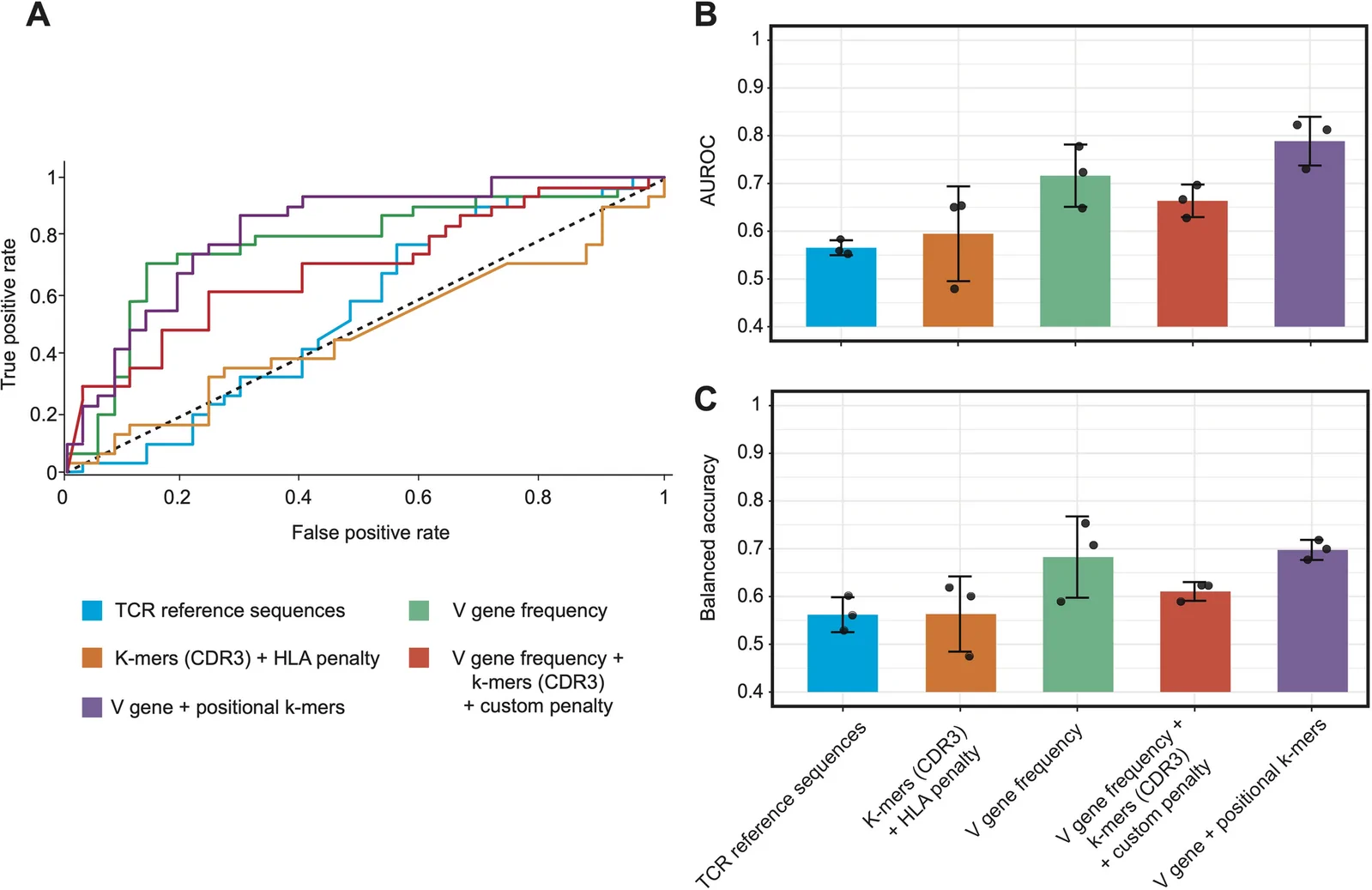
Model comparison for diagnosis classification based on naïve TCRs.** (A)** Receiver operating characteristic (ROC) curves for diagnosis classification using the naïve TCR dataset for the assessment set 2, representative of the three assessments. Dotted line indicates a baseline area under the curve (AUC) of 0.5. **(B)** AUROC for diagnosis classification performance using the naïve TCR dataset for the three assessment sets. **(C)** Balanced accuracy for diagnosis classification performance using the naïve TCR dataset for the three assessment sets. Individual datapoints represent the respective scores for each assessment split, bars represent the mean of the three splits ± one standard deviation (SD)

We first sought to determine if our models could successfully classify naïve TCR repertoires based on diagnosis (CeD or control) using supervised machine learning. After training on labeled datasets, our analysis revealed that classification using a positional k-mer-based approach, which utilized CDR3 k-mer information together with V gene identity, gave a moderately high balanced accuracy. When assessed on the test sets the median balanced accuracy score was 0.699 for the three assessment splits (Fig. 2). Classification using a model relying solely on V gene frequencies was shown to have a similarly high accuracy, with a median balanced accuracy score of 0.707 for the three assessment splits. This observation demonstrates the importance of TRV gene information for diagnosis classification. The gluten-specific reference-based approach had the worst classification performance, and it did not appear to capture any disease-related signals. This lack of performance suggests that, in a naïve repertoire, the gluten-specific T cell receptor reference sequences are not sufficient for classification of celiac individuals from controls.

### CeD diagnosis classification is not possible when restricted to HLA-DQ2.5^+^ individuals

**Fig. 3:**
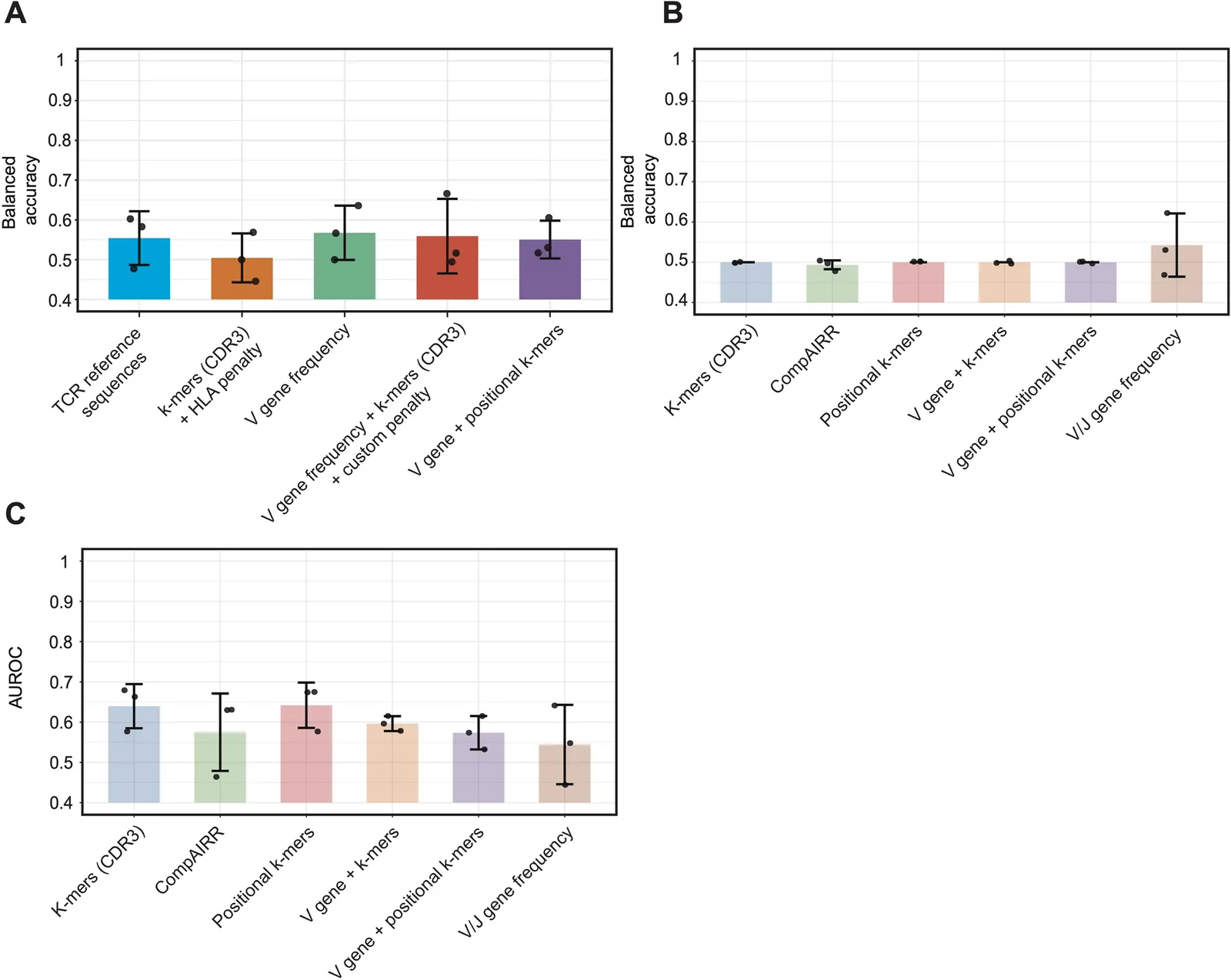
CeD diagnosis classification among HLA-DQ2.5^+^ individuals.** (A)** Balanced accuracy for diagnosis classification of HLA-DQ2.5^+^ individuals using the naïve TCR dataset for the three assessment sets. Models were trained on all naïve TCR repertoires. **(B)** Balanced accuracy for diagnosis classification using HLA-DQ2.5^+^ naïve TCR repertoires for the three assessment sets. Models were trained and assessed on only TCR repertoires of HLA-DQ2.5^+^ subjects. **(C)** AUROC for diagnosis classification using naïve TCR AIRR-seq repertoires of HLA-DQ2.5^+^ subjects for the three assessment sets. Models were trained and assessed on TCR repertoires of only HLA-DQ2.5^+^ subjects. Individual datapoints represent the respective scores for each assessment split, bars represent the mean of the three splits ± one SD

Following our finding that the naïve TCR repertoires could be classified by diagnosis with moderate accuracy and that previously, we have shown that distinct naïve TR gene usage between CeD individuals and controls could be attributed to the highly HLA-DQ2.5^+^ skewed composition of the CeD cohort compared to controls (Lindeman et al. 2026), we next sought to control for this allotype. When the models used in our diagnosis classification based on the TCR repertoires of all subjects were assessed on only HLA-DQ2.5^+^ individuals (defined as individuals with at least one HLA-DQ2.5 copy) (Fig. 3), the performance of the models was poor compared to our first analysis with balanced accuracy scores of around 0.5 across all models (Fig. 3A). This may have been because classification signals learned in the starting analysis were based on differences between HLA-DQ2.5 and other HLA-DQ allotypes and that the classifier had not captured further disease-related signals that could give better than random classification when stratified by DQ status. In support of this theory, a k-mer based model that penalized classification signals originating from HLA used for our diagnosis classification analysis did not appear to have any predictive capability (Fig. 2).

In addition to the stratified assessment, we also trained and assessed new models by selecting only the HLA-DQ2.5^+^ individuals in order to look for truly disease-associated signals not related to the HLA-DQ2.5 allotype (Fig. 3B, C). We used the TR V and J gene frequency, various k-mer-based models as well as a clonal overlap model to classify HLA-DQ2.5^+^ subjects by diagnosis (Fig. 3B, C). While k-mer based models – k-mers (CDR3) and positional k-mers - had slightly higher than random AUROC scores, there was large uncertainty in the performance estimation, and thus unclear if they captured any meaningful signal at all (Fig. 3C). None of the models were able to make accurate diagnosis calls as measured by balanced accuracy (Fig. 3B). While the HLA-DQ2.5-only analysis reduced the size of our dataset and therefore may have reduced the classification performance, the fact that we also did not see accurate classification from the stratified analysis adds further weight to the hypothesis that disease classification signals are based on HLA allotypes.

### HLA-DQ2.5^+^ individuals can be separated from HLA-DQ2.5^−^ individuals solely based on naïve TR V and J gene usage frequencies

**Fig. 4:**
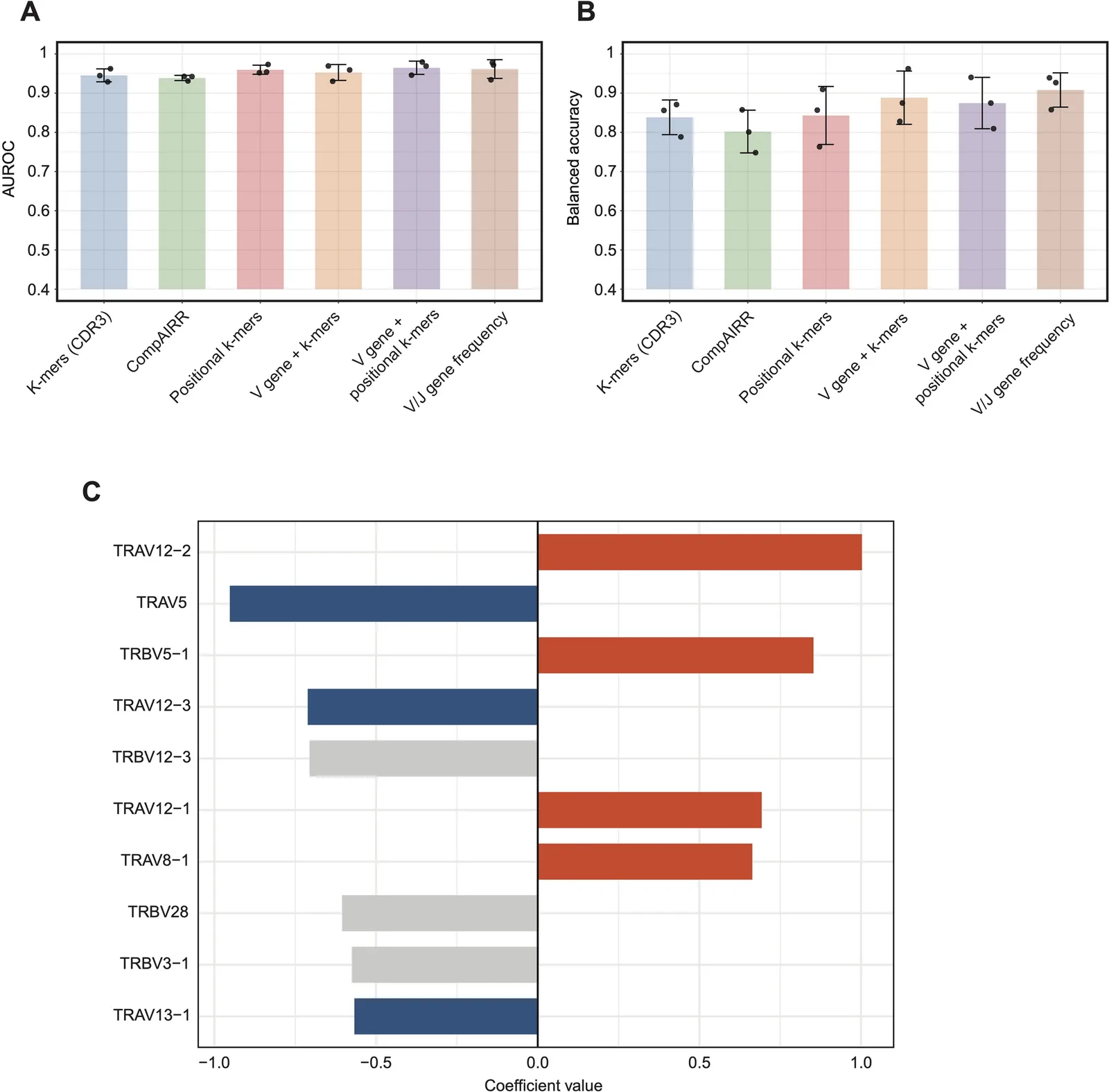
Classification of naïve TCR repertoires based on HLA-DQ allotype.** (A)** AUROC for HLA-DQ2.5 allotype classification using naïve TCR repertoires for the three assessment sets. **(B)** Balanced accuracy for HLA-DQ2.5 allotype classification using naïve TCR repertoires for the three assessment sets. Individual datapoints represent the respective scores for each assessment split, bars represent the mean of the three splits ± one SD. **(C)** Top 10 coefficients utilized by the V/J gene frequency model for assessment set 3, representative of the three assessments. Genes whose usage was previously positively associated with possession of the HLA-DQ2.5 allotype are colored red, genes whose usage was negatively associated with the HLA-DQ2.5 allotype are colored blue, and genes not previously reported as significantly associated with HLA-DQ2.5 are colored grey (Lindeman et al. 2026)

Since the diagnosis classification using the entire cohort showed moderately high accuracy when assessed on all individuals (Fig. 2), but not when assessed on HLA-DQ2.5^+^ individuals only (Fig. 3), we hypothesized that this classification was driven in large part by HLA allotypes, in particular HLA-DQ2.5. Therefore, using the entire cohort of TCR repertoires we next assessed the strength of classification signal that could be attributed to HLA-DQ allotypes and tested if our models could classify subjects into either HLA-DQ2.5^+^ or HLA-DQ2.5^−^ individuals. Our analysis revealed highly accurate classification with higher AUROC and balanced accuracy scores across all tested models compared to our diagnosis classification using all subjects (Fig. 4A, B). Models that utilized TR V/J gene frequency and k-mers with V gene information were among the most accurate classifiers, with median AUROC and balanced accuracy scores using the V/J gene frequency model of 0.972 and 0.927, respectively. This reflected a similar reliance on V gene information demonstrated by the diagnosis classification models in Fig. 2.

Based on the observation that TR V gene identity was shown to be an important feature for both accurate diagnosis and HLA allotype classification, we next investigated the TR V genes that were enriched in HLA-DQ2.5^+^ subjects. Previously, we have examined the TR gene usage frequencies of the same CeD individuals and controls (Lindeman et al. 2026). These previous results showed an association between TR gene usage and possession of the HLA-DQ2.5 allotype among controls. We integrated this information with the V and J gene coefficients which were demonstrated to be predictive of accurate HLA-DQ allotype classification in our machine learning analysis. TR V genes that previously were shown to have significantly higher usage among HLA-DQ2.5^+^ controls were well represented as positive coefficients identified using our machine learning models (Fig. 4C). Across the three assessment splits TRAV12-2 was listed as the top coefficient used for HLA-DQ2.5 classification in the V gene frequency model. The V gene frequency with positional k-mers model also consistently showed TRAV12-2 associated k-mers as top classification coefficients across the three assessment splits.

### CeD classification is not possible based on the naïve BCR repertoires

**Fig. 5:**
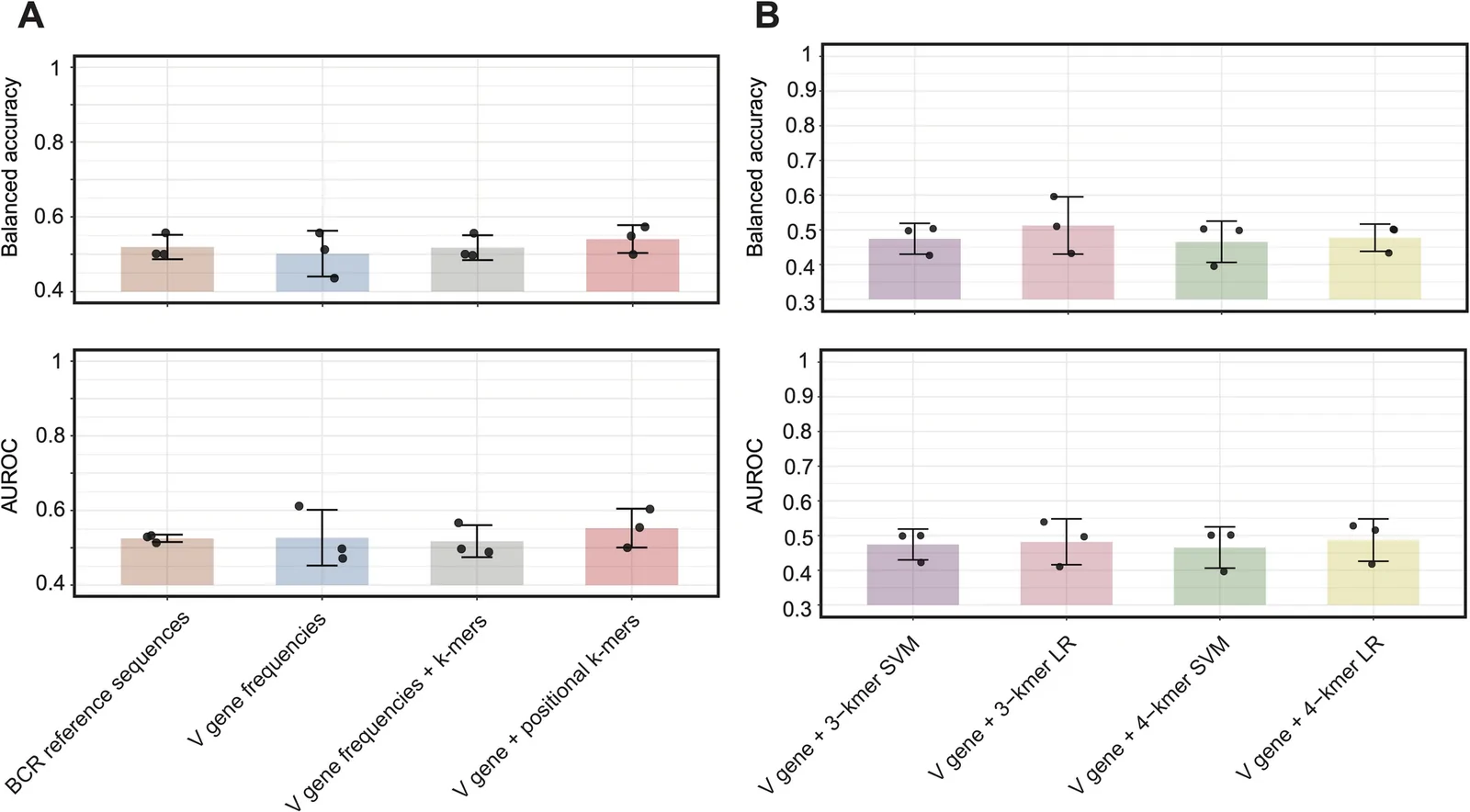
Diagnosis classification of naïve BCR repertoires.** (A)** Balanced accuracy (top) and AUROC (bottom) diagnosis classification performance using naïve BCR repertoires (BCR dataset 1) for the three assessment sets. **(B)** Balanced accuracy (top) and AUROC (bottom) diagnosis classification performance using the processed naïve BCR dataset 2a for the three assessment sets. Individual datapoints represent the respective scores for each assessment split, bars represent the mean of the three splits ± one SD

Using similar machine learning models employed for TCR, we sought to classify naïve BCR repertoires of the same subjects by diagnosis (BCR dataset 1). In contrast to our TCR analysis, none of the models provided accurate classifications for BCR dataset 1 with both AUROC and balanced accuracy performance scores indicating random classification (Fig. 5A). The naïve BCR repertoires of subjects with CeD previously have been stratified from those of controls using machine learning with high reported accuracy (Shemesh et al. 2021). As the exact parameters used in this study are unclear, we sought to assess whether any of our models could replicate their findings. To this end, we used two different versions of the dataset analyzed by Shemesh (BCR dataset 2a and 2b). One version utilized the already processed BCR AIRR-seq data used in the Shemesh publication as input to our machine learning models (BCR dataset 2a). For the second version, we obtained the raw BCR AIRR-seq data and processed it using a pipeline as similar to the one used for BCR dataset 1 as possible (BCR dataset 2b). None of our machine learning models resulted in better than random classification scores for the BCR dataset 2a (Fig. 5B**)** or for the BCR dataset 2b (Supplementary Fig. 1).

## Discussion

This study has interrogated the AIRRs of naïve T cells and B cells in subjects with CeD and disease controls. Our focus on the naïve repertoires was deliberate as distinct repertoires between patients and controls would indicate a genetic effect on AIRR development that is connected to disease risk. This approach contrasts with similar studies of effector or memory T cells and B cells that center on effects of stimulation and cell proliferation after encounter with antigen. We observed that the naïve CD4^+^ TCR repertoire allowed a moderately convincing classification of CeD subjects versus controls. The classification could be attributed to an effect of the HLA-DQ2.5 allotype, and accordingly, the HLA-DQ2.5 allotype could be distinguished with high accuracy using naïve TCR repertoires. By contrast to results of a previous study, the naïve BCR repertoire did not allow classification of CeD patients from controls.

Effector T cell populations in CeD have been well characterized and demonstrated to be specific to HLA-DQ2.2, DQ2.5 and DQ8-restricted gluten peptides (Jabri and Sollid 2017; Petersen et al. 2014, 2015, 2016; Qiao et al. 2014). These effector T cell populations have been examined by machine learning approaches in the past to accurately distinguish T cell repertoires belonging to CeD subjects from those of controls (Foers et al. 2021; Fowler et al. 2023; Yao et al. 2021). However, notably, to date no machine learning studies have considered the naïve CD4^+^ T cell repertoires in CeD. A recent large-scale TCR-HLA association study using both memory and naïve TCR sequences showed that by using memory TRB repertoire data, an individual’s HLA class I and II alleles could be predicted with high accuracy (Zahid et al. 2025). However, in this study when data from only naïve T cells was used, TRB sequences could not be assigned to HLA alleles, suggesting that HLA-associated TCRs arise primarily from post-thymic antigen-driven expansion. Our analysis allowed prediction of the HLA-DQ2.5 allotype on the basis of the naïve TCR repertoire. One explanation for this could be that our models additionally assess TRA repertoires, which we have recently shown are more strongly affected by HLA class II allotypes compared to TRB repertoires (Lindeman et al. 2026).

Across our TCR model configurations, the best-performing classifiers consistently relied on encodings that explicitly incorporate TCR V gene information, including V gene frequency features and V-gene–conditioned, position-aware k-mer representations. In contrast to models based solely on k-mers within the full CDR3 sequence, these encodings anchor sequence variation to germline V gene usage and, in the case of positional k-mers, to IMGT-defined alignment positions. This indicates that the predictive signal in naïve T-cell repertoires we studied is primarily driven by structured, germline-encoded features. These findings support X-ray crystal structural work on gluten-specific TCR interactions with HLA-DQ2.5 demonstrating multiple V-gene encoded CDR1 and CDR2 residues partaking in the molecular docking of the HLA-DQ2.5-restricted TCRs (Petersen et al. 2014). Moreover, our finding that the classifier based on TCR CDR3 k-mers was effective to partition HLA-DQ2.5^+^ versus HLA-DQ2.5^−^ individuals is in keeping with the observation that CDR3 residues also make contact with HLA-DQ2.5 in the X-ray crystal structures (Petersen et al. 2014). These results highlight the importance of accounting for HLA signal in machine learning disease classification as this signal could be interpreted as CeD-specific when it is indeed originating from HLA.

Given the moderately accurate performance of our diagnosis classification model using the TCR dataset, and that after stratifying our TCR repertoire cohort to only HLA-DQ2.5^+^ individuals all diagnosis predictive capability was abolished, this indicates that the CeD-predictive ability was a result of signals linked to HLA allotypes. Hence, the dominant signal detected in our study reflects *trans*-acting effects mediated by HLA rather than direct effects attributable to non-HLA *cis*-acting germline polymorphisms within antigen receptor loci themselves. In line with this, classification using the naïve BCR repertoires did not show any diagnosis predictive capability, and it failed to repeat the CeD diagnosis classification reported by Shemesh et al. 2021. The exact machine learning parameters used in the previous publication are however opaque, rendering exact replication difficult. Notwithstanding the failure to replicate previous results, BCR repertoire analysis has been reported to allow disease classification. In a recent study, BCR repertoires were analyzed by machine learning to classify patients into six different immune states with high reported accuracy. These BCR models incorporated class-switched B cell populations which could explain the impressive performance (Zaslavsky et al. 2025).

Our results support our recent finding that polymorphisms within the HLA loci shape the naïve TCR repertoire (Lindeman et al. 2026), consistent with thymic selection imprinting disease-relevant biases before antigen exposure. In the future, a larger HLA-balanced cohort could be utilized to assess the predictive capability of the naïve TCR repertoires for other HLA allotypes in addition to HLA-DQ2.5, and to disentangle HLA effects from disease status. Importantly, the absence of disease classification signal beyond HLA in the present study should not be interpreted as definitive evidence that non-HLA *cis*-acting germline polymorphisms lack relevance for CeD susceptibility. Although our findings suggest that such effects, if present, are likely modest relative to the dominant influence of HLA, alternative explanations must also be considered. Subtle repertoire-shaping effects may have been obscured by substantial inter-individual variability in naïve repertoires, limited statistical power after stratification to HLA-DQ2.5-positive individuals, or stochastic variability intrinsic to naïve lymphocyte repertoires. With the expansion of deep sequencing of the BCR and TCR loci and with identification of single nucleotide polymorphisms and structural variations (Engelbrecht et al. 2024; Gibson et al. 2023; Jana et al. 2025; Rodriguez et al. 2022, 2023; Zhang et al. 2021), appropriately sized genetic association studies should be able to provide further clarity as to whether BCR and/or TCR loci harbor genetic elements affecting the risk for CeD and other immune mediated diseases.

## Supplementary Information

Supplementary Material 1.

Supplementary Material 2.

## Data Availability

Both raw and processed BCR rep-seq dataset 1 and the TCR rep-seq dataset, and necessary metadata has been deposited at the European Genome-Phenome Archive (EGA) under accession numbers EGAS50000001881 and EGAS50000001882, under controlled access due to data protection. Data access is regulated through a data access agreement, and data usage is limited to studies aiming to investigate immunological mechanisms related to autoimmune diseases. Corresponding authors can be contacted with any inquiries related to data access. Data requests must be made through the EGA website and will be evaluated by the Data Access Committee (made up of L.M.S., I.L. and E.N-J.) within two weeks. Data analysis scripts can be made available by the authors upon reasonable request. The pre-processed BCR repertoire sequencing dataset 2 can be found at the European Nucleotide Archive (ENA), under accession number PRJEB26509.
