# Supplementary material for: Naïve adaptive immune receptor repertoires in celiac disease assessed by machine learning; impact of the HLA-DQ2.5 allotype on the TCR repertoire
Source: Immunogenetics. 2026 Aug 27;78(1):20. doi: 10.1007/s00251-026-01412-3 (PMC13518406; doi:10.1007/s00251-026-01412-3)
Supplement: Supplementary file 1 [file 251_2026_1412_MOESM1_ESM.docx]

**Supplementary materials & methods**

Naïve adaptive immune receptor repertoires in celiac disease assessed by machine learning; Impact of the HLA-DQ2.5 allotype on the TCR repertoire

Aengus Officer, Ida Lindeman, Milena Pavlovic, Christin Lund-Andersen, Eivind Ness-Jensen, Geir Kjetil Sandve, Ludvig M. Sollid

\##

**TCR repertoire analysis feature encodings**

Three analyses using naïve TCR repertoire data were performed: (1) Classification by diagnosis, (2) Classification by diagnosis within HLA-DQ2.5^+^ individuals, (3) HLA-DQ2.5 classification.

CDR3 sequences were represented using 3-mer amino-acid motif frequencies. For each repertoire, the relative frequency of each possible 3-mer was calculated across all unique sequences. Several variants of this encoding were used:

*Continuous k-mer encoding:* 3-mer motifs were extracted directly from the amino-acid CDR3 sequence.

*IMGT-aligned k-mer encoding:* 3-mers were extracted from CDR3 sequences aligned according to IMGT positional numbering, preserving positional information across sequences.

*V-gene conditioned k-mers:* k-mer features were stratified by V-gene usage, allowing motif frequencies to vary conditional on the TRBV gene.

*V-gene IMGT-conditioned k-mers:* k-mers were conditioned on both V-gene identity and IMGT positional alignment. In all k-mer encodings, feature values were expressed as relative frequencies within each repertoire. For some encodings, features were scaled to unit variance before model fitting.

Gene-usage encoding: Repertoires were encoded using the relative frequencies of V-gene or V- and J-gene usage. These features capture repertoire biases arising from V(D)J recombination and thymic selection.

Reference-sequence encodings: To assess whether repertoires contained sequences previously associated with gluten recognition, repertoires were compared with a curated reference set of public gluten-reactive TCR sequences. Two encodings were derived:

*Matched-sequence presence:* Binary features indicating the presence of exact matches to reference sequences.

*Matched-sequence summary:* A single feature representing the total number of reference sequence matches within the repertoire. Sequence matches required exact CDR3 identity.

Shared-clonotype abundance encoding: For TCR analyses (2) and (3), TCR repertoire similarity across individuals was also evaluated using the CompAIRR algorithm. This method identifies sequences shared across repertoires and quantifies their relative abundance.

**TCR analysis machine-learning models**

Three different classifier types were used:

*Logistic regression:* Most classification models used logistic regression with L1 regularization (LASSO). For a repertoire feature vector $x$, the probability of class membership was modeled as

$$P(y=1\mid x)=\frac{1}{1+e^{-(\beta_{0}+\beta^{T}x)}}$$

Model parameters were estimated by minimizing the penalized logistic loss

$$L(\beta)=-\mathcal{l}(\beta)+\lambda\mid\mid\beta\mid\mid_{1}$$

where $\mid\mid\beta\mid\mid_{1}$denotes the L1 norm of the coefficient vector and $\lambda$ controls the strength of regularization.

The inverse regularization parameter $C=1/\lambda$ was selected by grid search over the values:

$$C\in\{0.01,\text{ }0.1,\text{ }1,\text{ }10,\text{ }100\}.$$

*Logistic regression with selective regularization:* For the model in analysis 1 that incorporated HLA metadata (“k-mers + HLA”), we used a modified logistic regression in which regularization was applied to sequence-derived features but not to the HLA covariate. This approach allowed the model to account for the contribution of HLA genotype while regularizing the high-dimensional sequence-derived feature space and thereby assessing whether repertoire features provided predictive signal beyond HLA as described previously (De Bin et al., 2020). For the “V gene frequency + k-mers + custom penalty”, we employed model regularization on the k-mers, but not on the gene covariate as we wanted the overall effect of V-genes to be captured in the gene coefficient rather than being spread across multiple V-gene-specific k-mer coefficients.

*Probabilistic binary classifier:* Shared-sequence abundance features derived using CompAIRR were evaluated using a probabilistic binary classifier optimized via iterative likelihood maximization. Model fitting was performed for up to 50,000 iterations with an update rate of 0.01. Two sequences were considered an exact match if they had matching V- and J-gene assignments and CDR3 amino acid sequences. We did this in a similar manner to the approach in Emerson et al., 2017, where we used the set of all receptor sequences present in any repertoire as our features, and then encoded each repertoire based on which of these receptor sequences were present in a given repertoire using a method detailed elsewhere (Rognes et al., 2022) to compute this efficiently.

**TCR analysis model evaluation and hyperparameter optimization**

Model hyperparameters were optimized using grid search with cross-validation.

During model selection:

- 5-fold cross-validation was used for analyses (2) and (3).
- 3-fold cross-validation was used for analysis (1).

Models were trained on 70% of the data and evaluated on the remaining 30%. For final assessment, three independent random splits of the dataset were performed.

**TCR analysis encodings evaluated for each classification task**

The following feature encodings were evaluated in each analysis.

*Analysis 1: Classification by diagnosis*

Five models were evaluated:

1. V-gene frequency encoding with logistic regression.
2. V-gene conditioned CDR3 k-mer encoding with logistic regression.
3. CDR3 k-mer encoding combined with HLA status using logistic regression with selective regularization.
4. Exact matches to public gluten-reactive TCR sequences with logistic regression.
5. Logistic regression model trained on combined V-gene usage frequencies and CDR3 k-mer features, with L1 regularization applied to k-mer features while V-gene-derived features are exempt from penalization.

*Analysis 2: Classification by diagnosis within HLA-DQ2.5 individuals*

Six models were evaluated:

1. Continuous CDR3 k-mer encoding.
2. IMGT-aligned CDR3 k-mer encoding.
3. V-gene conditioned k-mer encoding.
4. V-gene and IMGT conditioned k-mer encoding.
5. V- and J-gene frequency encoding.
6. Shared-sequence abundance encoding using CompAIRR with a probabilistic classifier.

*Analysis 3: HLA-DQ2.5 classification*

The same six repertoire encodings used in analysis 2 were evaluated to classify individuals with HLA-DQ2.5 from individuals carrying other HLA-DQ types.

**BCR repertoire analysis**

We evaluated a range of machine learning models, including logistic regression (LR) and support vector machines (SVM), applied to multiple BCR repertoire encodings implemented in ImmuneML. These encodings employed gene usage features, k-mer based sequence representations and reference-matching approaches.

*BCR dataset 1*

Reference sequence-based encodings: each repertoire was represented by its similarity to a predefined set of reference BCR sequences. A sequence was considered a match if it shared V- and J-gene assignments and a CDR3 amino acid sequence matching the reference sequence, allowing for up to one amino acid mismatch to account for somatic hypermutation.

Gene frequency encodings: based on the relative usage of V genes across each repertoire.

Position-aware k-mer encodings: derived from CDR3 amino acid sequences, including 3-mers indexed with IMGT positional information and V-gene context, enabling joint modeling of local sequence structure and germline gene usage.

Gene frequency and k-mer encoding: combining k-mer features and V-gene frequencies, allowing simultaneous modeling of sequence-level motifs and gene-level effects within a unified feature space.

*BCR dataset 2*

B cell receptor repertoires were encoded using V-gene–conditioned k-mer frequency representations derived from CDR3 amino acid sequences. Two feature sets were constructed using k = 3 and k = 4, which integrates local sequence composition with V-gene identity. Additionally, V- and J-gene frequency encodings were also used.

Support vector machines and logistic regression models were trained on each encoding variant. Repertoires were filtered to IMGT CDR3 amino acid sequences with a minimum length of five amino acids, and only productive sequences were retained.

**Cross validation**

To evaluate performance, datasets were separated into training, validation and independent test subsets. Either three or five-fold cross-validation was performed to test how well the models generalized by training the models on the training split, choosing model hyper-parameters on the validation split and getting an unbiased performance estimate of each model on the assessment split.

**Supplementary materials & methods references**

De Bin, R., Boulesteix, A.-L., Benner, A., Becker, N., & Sauerbrei, W. (2020). Combining clinical and molecular data in regression prediction models: Insights from a simulation study. *Briefings in Bioinformatics*, *21*(6), 1904–1919. https://doi.org/10.1093/bib/bbz136

Emerson, R. O., DeWitt, W. S., Vignali, M., Gravley, J., Hu, J. K., Osborne, E. J., Desmarais, C., Klinger, M., Carlson, C. S., Hansen, J. A., Rieder, M., & Robins, H. S. (2017). Immunosequencing identifies signatures of cytomegalovirus exposure history and HLA-mediated effects on the T cell repertoire. *Nature Genetics*, *49*(5), 659–665. https://doi.org/10.1038/ng.3822

Rognes, T., Scheffer, L., Greiff, V., & Sandve, G. K. (2022). CompAIRR: Ultra-fast comparison of adaptive immune receptor repertoires by exact and approximate sequence matching. *Bioinformatics*, *38*(17), 4230–4232. https://doi.org/10.1093/bioinformatics/btac505
