# Supplementary material for: Naïve adaptive immune receptor repertoires in celiac disease assessed by machine learning; impact of the HLA-DQ2.5 allotype on the TCR repertoire
Source: Immunogenetics. 2026 Aug 27;78(1):20. doi: 10.1007/s00251-026-01412-3 (PMC13518406; doi:10.1007/s00251-026-01412-3)
Supplement: Supplementary file 2 [file 251_2026_1412_MOESM2_ESM.docx]

**Supplementary figures**

Naïve adaptive immune receptor repertoires in celiac disease assessed by machine learning; Impact of the HLA-DQ2.5 allotype on the TCR repertoire

Aengus Officer, Ida Lindeman, Milena Pavlovic, Christin Lund-Andersen, Eivind Ness-Jensen, Geir Kjetil Sandve, Ludvig M. Sollid

\##

***Supplementary figure 1. Diagnosis classification using BCR dataset 2b. (A)*** *Balanced accuracy and* ***(B)*** *AUROC diagnosis classification performance using the raw subset of BCR dataset 2b for the three assessment test sets. VJ frequency-based models were either normalized to a relative frequency or measured as absolute counts. Individual datapoints represent the respective scores for each assessment split, bars represent the mean of the three splits ± one standard deviation.*

**A**

Distribution of CeD individuals in TCR analysis (1) by HLA label

**B**

Distribution of HC individuals in TCR analysis (1) by HLA label

**C**

Distribution of individuals in TCR analysis (2) by diagnosis label

**D**

Distribution of CeD individuals in TCR analysis (3) by HLA label

**E**

Distribution of HC individuals in TCR analysis (3) by HLA label

***Supplementary figure 2. Distribution of individuals by primary label across TCR repertoire analyses.*** *The numbers of individuals used in each of the three training and three test sets for each of the TCR analyses are shown.*
